# Nipple-sparing mastectomy: external validation of a three-dimensional automated method to predict nipple occult tumour involvement on preoperative breast MRI

**DOI:** 10.1186/s41747-019-0108-3

**Published:** 2019-08-07

**Authors:** Marta D’Alonzo, Laura Martincich, Agnese Fenoglio, Valentina Giannini, Lisa Cellini, Viola Liberale, Nicoletta Biglia

**Affiliations:** 1 Obstetrics and Gynaecology Unit, Umberto I Hospital, Corso Turati, 62, 10128 Turin, TO Italy; 2 Unit of Radiology, ICandiolo Cancer Institute, FPO-IRCCS, Strada Provinciale, 142 - KM 3.95, 10060 Candiolo, TO Italy; 3 Department of Radiology, Umberto I Hospital, Corso Turati, 62, 10128 Turin, TO Italy; 4 Department of Surgical Sciences, University of Turin, Turin, TO Italy

**Keywords:** Algorithm, Breast neoplasms, Magnetic resonance imaging, Mastectomy, Nipple

## Abstract

**Background:**

Preoperative evaluation of nipple-areola complex (NAC) tumour involvement is crucial to select patients candidates for nipple-sparing mastectomy. Our aim was to validate a previously developed automated method able to compute the three-dimensional (3D) tumour-to-NAC distance (the most predictive parameter of nipple involvement), using magnetic resonance imaging (MRI) datasets acquired with a scanner and protocol different from those of the development phase.

**Methods:**

We performed a retrospective analysis of 77 patients submitted to total mastectomy and preoperatively studied with MRI. The new method consisted of automated segmentation of both NAC and tumour and subsequent computation of the 3D distance between them; standard manual two-dimensional segmentation was independently performed. Paraffin-embedded section examination of the removed NAC was performed to identify the neoplastic involvement. The ability of both methods to discriminate between patients with and without NAC involvement was compared using receiver operating characteristic (ROC) analysis.

**Results:**

The 3D tumour-to-NAC distance was correctly computed for 72/77 patients (93.5%); tumour and NAC segmentation method failed in two and three cases, respectively. The diagnostic performance of the 3D automated method at best cut-off values was consistently better than that of the 2D manual method (sensitivity 78.3%, specificity 71.4%, positive predictive value 87.5%, negative predictive value 56.3%, and AUC 0.77 versus 73.9%, 61.2%, 47.2%, 83.3%, and 0.72, respectively), even if the difference did not reach statistical significance (*p* = 0.431).

**Conclusions:**

The introduction of the 3D automated method in a clinical setting could improve the diagnostic performance in the preoperative assessment of NAC tumour involvement.

## Key points

- The distance between tumour and nipple-areola complex (NAC) on magnetic resonance imaging (MRI) is the best predictor of occult nipple involvement, essential for planning nipple-sparing mastectomy.
- A novel three-dimensional (3D) automated method for computing tumour-to-NAC distance on MRI datasets was developed.
- This new method was compared with two-dimensional (2D) manual measurement on an external dataset, i.e. different from that used in the development phase.
- In the external validation, the 3D automated method showed a sensitivity and a specificity higher than those of 2D manual measurement, even though not reaching the statistical significance.

## Background

Since its introduction, nipple-sparing mastectomy (NSM) has become a frequent surgical option for both prophylactic and therapeutic indications.

The shift towards more conservative types of mastectomy, which began in the early 1990s with the introduction of the skin-sparing mastectomy [1], led to a revolutionary concept in oncology, with “oncoplastic” surgery gaining increasing popularity. Skin-sparing mastectomy (SSM) allows for an immediate implant-based reconstruction of the breast. However, the frequent dissatisfaction for nipple remodelling [2], combined with the evidence of the low rate of tumour involvement of the nipple-areola complex (NAC) [3, 4], made it possible to offer the preservation of the nipple and areola to selected breast cancer patients [5]. Thus, NSM got a foothold all over the world, thanks to its advantage in terms of patient satisfaction [6], along with the reassuring results of several retrospective studies and meta-analysis on its oncological safety [7–9].

In patients who might be offered NSM, the preoperative assessment of NAC tumour involvement is crucial to optimise the surgical planning. Some predictive models have been developed [10, 11] to provide a probability score of NAC occult involvement, by evaluating several tumour characteristics [12]. According to those studies, the tumour-to-NAC distance measured using MRI rather than mammography has proven to be the key predictor of occult nipple involvement [13, 14].

Despite the available evidence, this data is often difficult to obtain, primarily because the analysis of MRI requires interpretation by experienced breast radiologists and secondly because it is subject to operator-dependent variability, thus resulting in a difficult standardisation of the parameter [15]. Furthermore, tumour-to-NAC distance is usually measured two-dimensionally on maximum intensity projection (MIP) images [14], leading to the loss of important spatial information, and not infrequently giving results inconsistent with the clinical situation.

To overcome these issues, recently Giannini et al. [16] developed a fully automated method for breast dynamic contrast-enhanced (DCE) MRI, able to detect both tumour lesions and NAC, and to compute the three-dimensional (3D) distance between them, providing an objective and reproducible final measurement. This algorithm outperformed the results obtained with MRI manual distance evaluation, by reaching sensitivity and specificity of 72% and 80%, respectively, in detecting NAC involvement, with a negative predictive value of 89%. These promising results were obtained using a single-centre dataset. If validated, this method could help in preoperatively identifying a higher number of patients who may benefit from NSM with a consequent impact on the choice of the optimal oncoplastic approach (i.e. one-stage versus two-stage surgery).

The aim of the current study is to assess the automated three-dimensional (3D) tumour-to-NAC distance calculation with the algorithm proposed by Giannini et al. [16] for the preoperative evaluation of candidates for NSM and to validate it on a large image dataset acquired in a different institution using a different MRI scanner and acquisition protocol.

## Methods

### Patient selection

Patients who received total or SSM for breast cancer between January 2010 and May 2016 at the Academic Breast Unit of the Umberto I Hospital, University of Turin, were considered eligible for the present study.

**Fig. 1:**
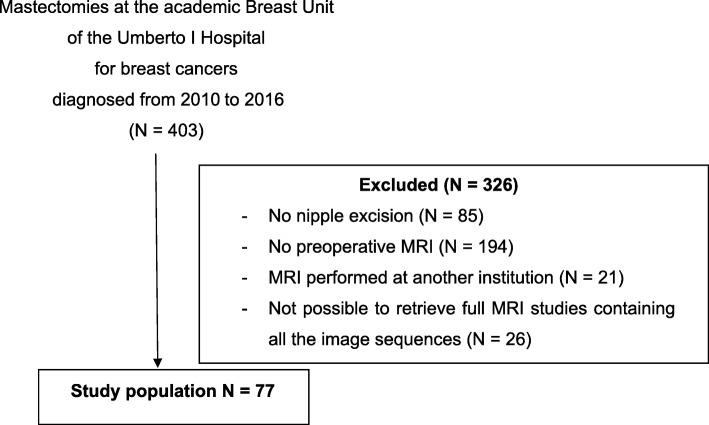
Patient selection diagram. *N* number, *MRI* magnetic resonance imaging

Data from 403 mastectomy specimens were retrieved. Patients who had the nipple preserved were excluded, based on the lack of definitive data on histological examination of the terminal ducts. In addition, patients who had not been studied with preoperative breast MRI, including T2-weighted images and dynamic study, were excluded. Metastatic disease and age at surgery were not considered as exclusion criteria. Of the 124 cases initially selected, 103 had undergone MRI examination at our institution and MRI data were therefore effectively accessible. Among these, it was possible to retrieve in the archives of the Department of Radiology 81 full MRI studies. Of these, four did not contain all the image sequences and were excluded. Overall, 77 cases were available for the analysis. A study flow diagram is provided in Fig. 1.

Written informed consent to the anonymous use of their clinical and instrumental data for scientific purposes is signed by all the patients treated at our institution.

### MRI

Breast MRI was performed using a 1.5-T scanner (Ingenia, Philips Medical Systems, Best, The Netherlands) and a dedicated phased-array 16-channel coil, with the patient in the prone position, following the recommended requirements [17]. In particular, the DCE MRI acquisition was performed using a T1-weighted high-resolution isotropic volume examination (THRIVE) sequence (voxel 1 × 1 × 1 mm; acquisition matrix 280 × 339; temporal resolution 70 s) acquired before and six times after intravenous contrast agent administration (0.1 mmol/kg of Gadobutrol, Gadovist^@^ Bayer) at a flow rate of 2 mL/s, followed by saline flushing of 20 mL at a flow rate of 2 mL/s. Table 1 shows the differences between MRI scanner, devices, and technical MRI acquisition parameters used in the current study and those used for the automated software developed by Giannini et al. [16].

**Table 1:** Differences between scanner, devices, and technical parameters of dynamic contrast-enhanced acquisition used in the current study and those used in the work by Giannini et al. [16]

		Current study	Giannini et al. [16]
Equipment		1.5-T Ingenia; Philips Medical Systems, Best, The Netherlands	1.5-T HDx Signa Excite, GE HealthCare Milwaukee, WI, USA
Coil		Phased-array 16-channel	Phased 8-channel coil
DCE	Slice thickness (mm)	3	2.6
	Acquisition plane	Axial	Axial/sagittal
	Repetition time (ms)	5.1	5.5/4.8
	Echo time (ms)	2.5	2.6/1.9
	Flip angle	10°	10°
	Field of view	According to breast volume	According to breast volume
	Acquisition matrix	340 × 340	416 × 416/416 × 256
	Pixel size (mm^2^)	0.7865 × 0.7865	Pixel 0.625 × 0.625
	Temporal resolution (s)	70	90
T2-weighted	Slice thickness (mm)	3	3
	Acquisition plane	Sagittal	Sagittal
	Repetition time (ms)	2000	3360
	Echo time (ms)	209	70
	Flip angle	90°	90°
	Field of view	According to breast volume	According to breast volume
	Acquisition matrix	340 × 340	416 × 256
	Pixel size (mm)	0.8536 × 0.8536	0.4297 × 0.4297

### Measurement of the tumour-to-NAC distance (manual method)

The tumour-to-NAC distance was defined as the minimum distance between the base of the NAC (considered as the line under the nipple passing along the areola plane) and the nearest margin of tumour focus. As described in a previous study [14], the tumour-to-NAC-distance was both manually measured by electronic calipers on axial maximum intensity projection (MIP) images by breast radiologist with a 10-year experience in breast MRI and automatically obtained by processing images with the automated algorithm. Both of these measurements were evaluated for their ability to predict the likelihood of NAC occult involvement.

### Measurement of the tumour-to-NAC distance (automated method)

**Fig. 2:**
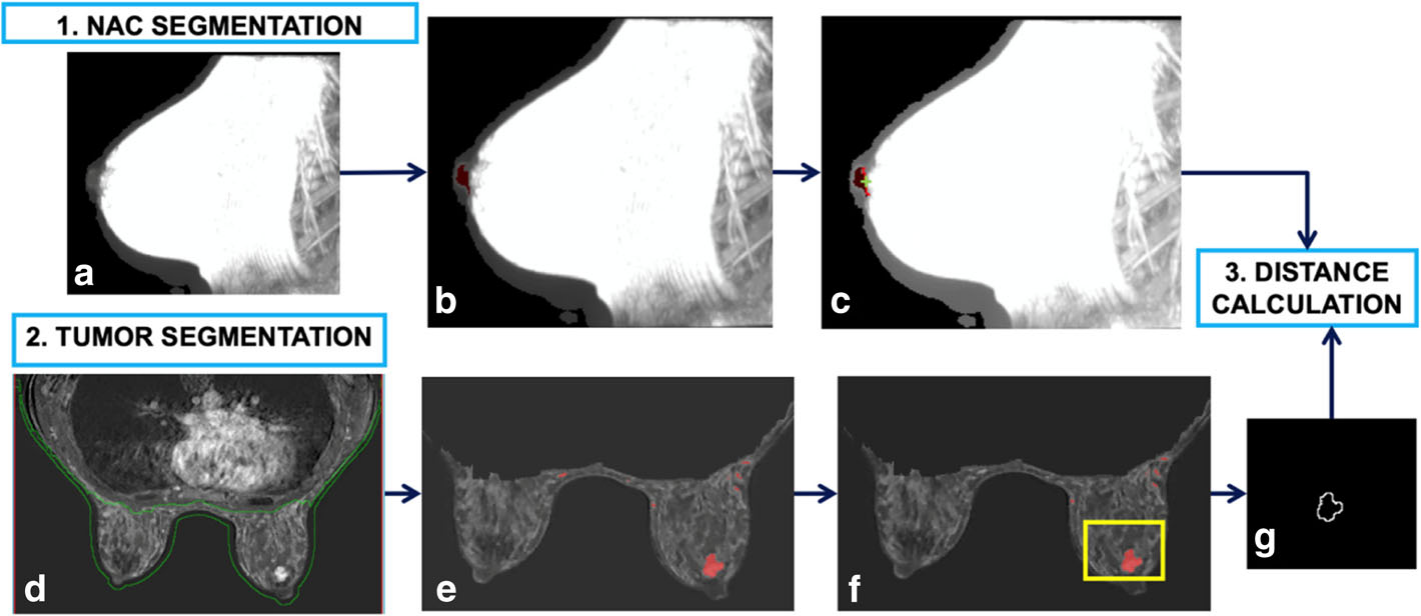
Pipeline of the segmentation algorithm. **a** Maximum intensity projection (MIP) of the T2-weighted dataset. **b** Segmentation of the nipple-areola complex (NAC) superimposed to the T2-weighted MIP. **c** B-spline curve that represents the base of the NAC superimposed to the T2-weighted MIP (the point used to compute the distance is highlighted by using a green cross). **d** Segmentation of the breasts superimposed to the second subtracted contrast-enhanced frame. **e** Segmentation results before applying the false-positive reduction step. **f** Segmentation results after applying the false-positive reduction step (the yellow box represent the region that the radiologist selected as tumour). **g** The outer edge of the selected tumour

The automated calculation of 3D tumour-to-NAC distance was performed using the previously described algorithm [16] which consists of three main steps: (a) nipple segmentation, (b) lesion segmentation, and (c) measuring of tumour-to-NAC distance. The first phase is the segmentation of the nipple (Fig. 2a–c) which uses the axial images of the breast to identify the position of the nipple as the most anterior point of the body region represented on the image. A “growing region” algorithm is then applied, which selects some seeds (pixels) of the nipple in T2-weighted MIP image (Fig. 2a) and segments the entire NAC area using a threshold of ± 50% of the seed voxel intensity (Fig. 2b). The so-called mask of the nipple is then obtained, then a third order B-spline interpolation [18] is applied to the mask in order to attenuate the irregularities and to reproduce the base of the NAC (Fig. 2c). The B-spline curve is a curve constructed by joining several segments that constitute a continuously broken line formed by the pixels that delimit the inner edge of NAC mask, whose trend is the function of the number and position of the control points that affect the curve tracts in their own. The second phase is the segmentation of the lesion (Fig. 4), which is based on an algorithm previously developed and described by Giannini et al. [15, 19]. Briefly, the algorithm segments the breasts area (Fig. 2d) and extracts the contrast-enhanced regions using the post-contrast images, previously normalised with the signal intensity of the mammary vessels, automatically segmented by the system (Fig. 2e). Once the tumour and the NAC regions are detected, some criteria are applied to reduce the number of false positives (i.e. vessels, image artefacts) (Fig. 2f). Finally, the radiologist manually selects the most anterior lesion (the one closer to the nipple), as shown in the yellow box in Fig. 2f. This is the only manual step of the procedure. Once the NAC and the tumour lesion are segmented, the 3D Euclidean distance between the central point of the B-spline interpolation of the internal border of the NAC (the green cross in Fig. 2c and the closest point of the border of the lesion in Fig. 2g).

**Fig. 3:**
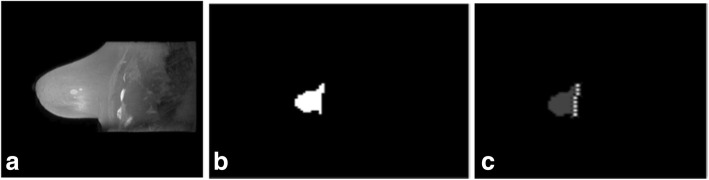
Segmentation of the nipple and final mask of the nipple-areola complex. **a** MIP image (maximum intensity projection) performed on T2-weighted image and seed selection. **b** Segmentation of the nipple (region growing technique) and final mask of the nipple areola complex. **c** Interpolation of points on nipple base

**Fig. 4:**
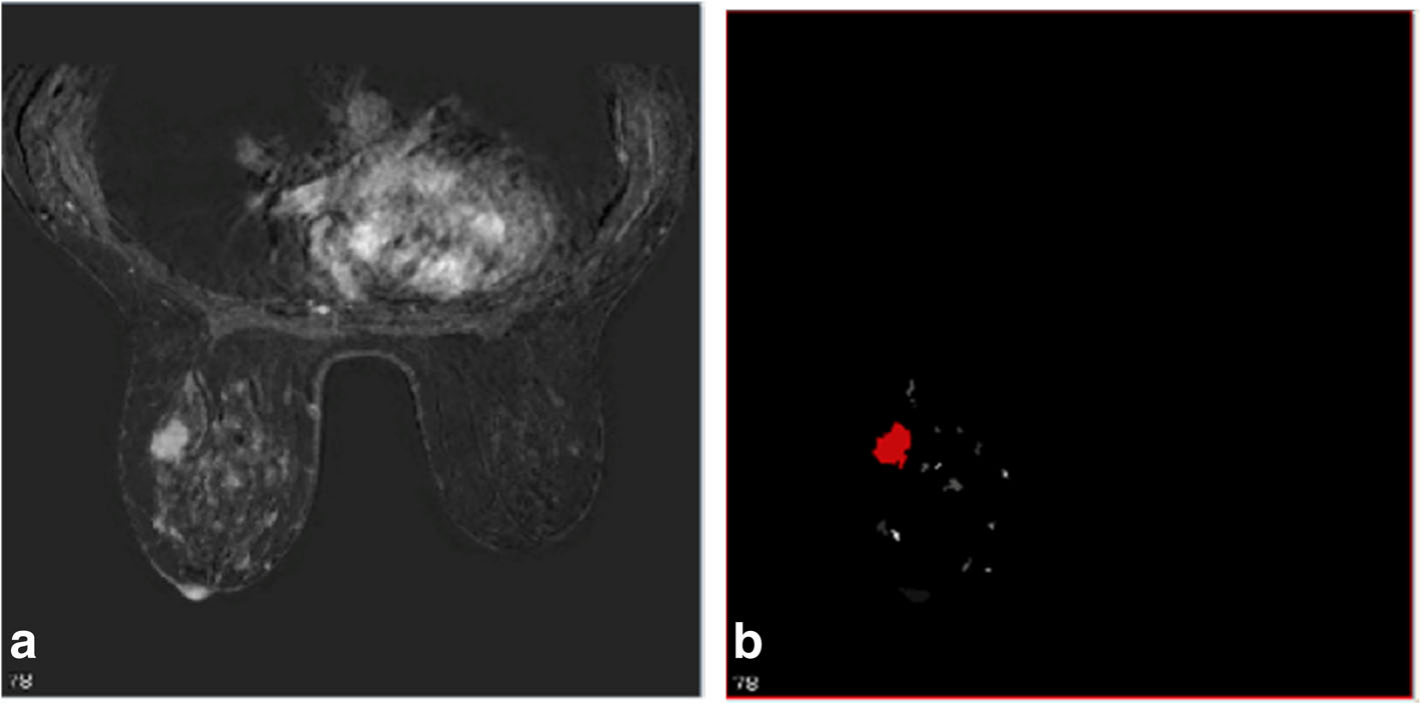
Segmentation of the tumour and final mask. **a** Axial image of the most representative slice. **b** Final mask of segmentation of the tumour, selected by the radiologist

Average execution time was 8 min to segment the tumour lesions, 2 min to segment the nipples, and 1 min to compute the 3D tumour-to-NAC distance. The execution time was measured on a computer equipped with a CPU Intel Core i7 940 Quad Core @#2.93 GHz architecture and 8 GB RAM. Figures 3 and 4 show other examples of NAC and tumour segmentation.

### Pathological examination of the nipple

The pathological sections were used as the reference standard for the diagnostic performance of the two methods of calculation of tumour-to-NAC distance on MRI. Final histological reports were reviewed by a pathologist with more than 10 years of experience in breast pathology. NAC involvement was defined by the presence of invasive ductal or lobular carcinoma and/or ductal carcinoma in situ (DIN1c–DIN3) within the retro-areolar margin (2–3 mm below the areola-nipple junction). Lobular carcinoma in situ/lobular intraepithelial neoplasia (LIN1–LIN3) was not considered as malignant lesions.

### Statistical analysis

Diagnostic performances of both automated and manual methods to compute tumour-to-NAC distance were recorded: sensitivity, specificity, positive predictive value (PPV), negative predictive value (NPV), and overall diagnostic accuracy.

True positives were defined as those cases in which the automated or manual method provided a tumour-to-NAC distance lower than the chosen cut-off and the pathologic report confirmed tumour NAC involvement. False positives were defined as those cases in which the automated or manual method provided a tumour-to-NAC distance lower than the chosen cut-off, but the pathologic report showed the absence of tumour NAC involvement. True negatives were defined as those cases in which the automated or manual method provided a tumour-to-NAC distance equal or higher than the chosen cut-off and the pathologic report confirmed the absence of tumour NAC involvement. False negatives were defined as those cases in which the automated or manual method provided a tumour-to-NAC distance equal or higher than the chosen cut-off and the pathologic report showed tumour NAC involvement.

When 95% confidence intervals were provided, they were calculated according to the binomial distribution.

Receiver operating characteristic (ROC) curves were obtained, and the best cut-off value was searched for optimal balance between sensitivity and specificity for both methods. Different cut-off values were also considered, according to those used in the literature (i.e. 5, 10, 20, and 30 mm). A *p* value < 0.05 was considered as statistically significant.

Statistical analysis was performed using SPSS, version 24 for Windows (IBM Corporation, Armonk, NY, USA), and MedCalc, version 13.2.2.0 (MedCalc Software, Ostend, Belgium). All calculations were performed on both softwares.

## Results

From the raw series of 77 patients with 77 tumour lesions, 2 were excluded because the tumour lesion was not correctly detected by the algorithm (97% per-lesion sensitivity, 95% confidence interval 91–100%), 2 because of an error in the segmentation or malposition of the nipple, and 1 because the possible NAC involvement at the final pathology examination was not specified in the report. The two false-negative tumour lesions were one invasive ductal carcinoma, with a maximum diameter of 7 mm, and one ductal carcinoma in situ, with a maximum diameter of 23 mm, both of them with a poor contrast enhancement on the axial DCE sequence.

Thus, we tested the algorithm on both nipples for each of the 72 patients, and the nipples properly segmented were 141/154 (sensitivity 92%, 95% confidence interval 86–95%). Of the 72 patients with evaluated tumour-to-NAC distance, 23 (32%) showed tumour infiltration of the nipple at final pathology.

**Fig. 5:**
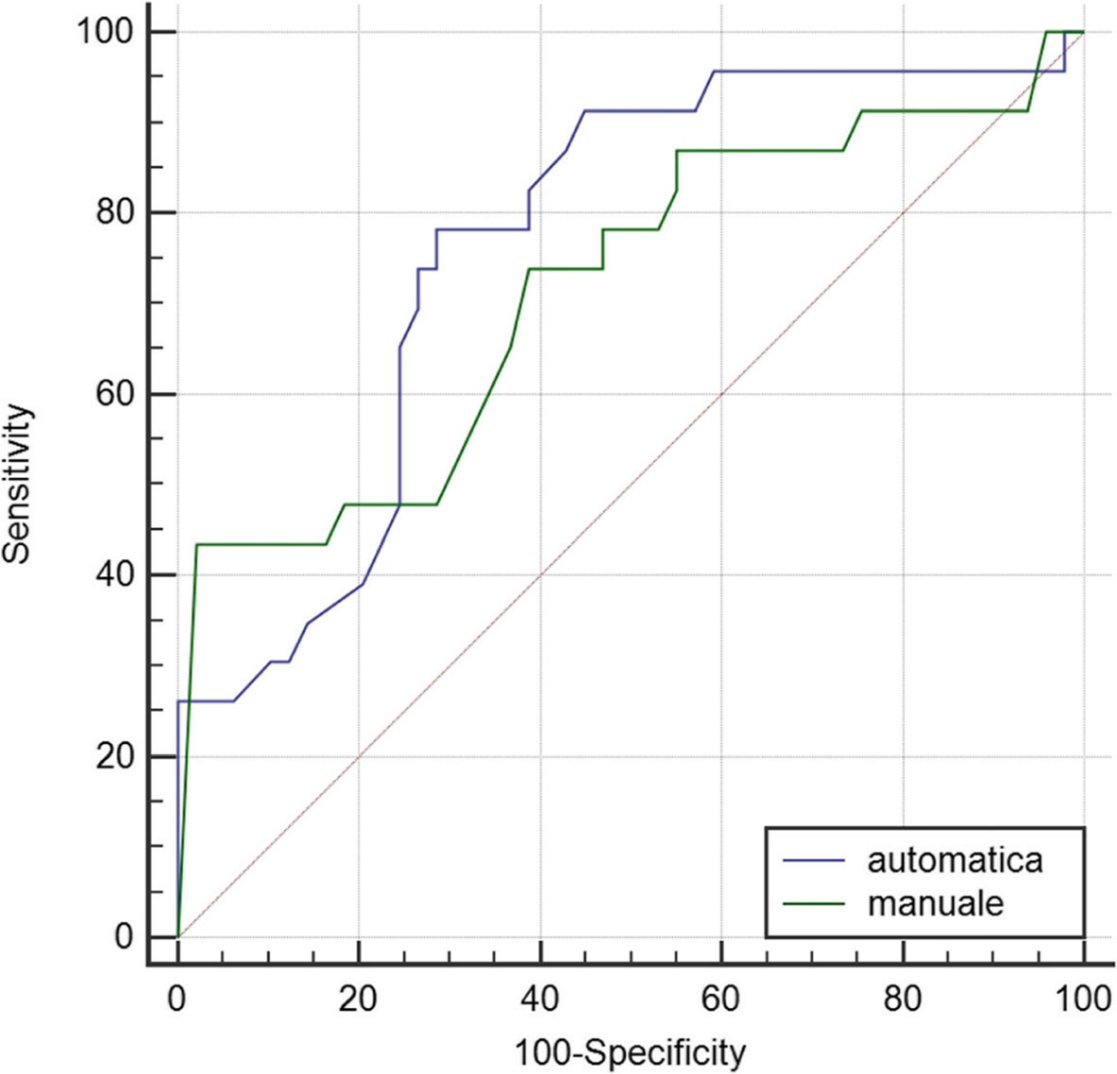
Comparison between receiver operating characteristic curves for the performance of automatic (blue) and manual (green) tumour-to-nipple-areola complex distance

The ROC curves obtained for both 2D manual and 3D automated tumour-to-NAC distances are shown in Fig. 5. Overall, the diagnostic performance of the automated tumour-to-NAC distance (area under the curve [AUC] = 0.76) was slightly superior to manual axial distance (AUC = 0.72). However, the difference between them was not statistically significant (*p* = 0.431).

Sensitivity, specificity, PPV, NPV, and accuracy calculated at different cut-off values for the two methods are shown in Table 2. The best cut-off, which expresses the best balance between sensitivity and specificity, is 21 mm for the manual method and 30 mm for the automated system. Overall, the automated system reached the highest accuracy in predicting the NAC involvement, in particular, for the best cut-off and also for the cut-off at 10 mm. Only when comparing the data for the cut-off of 5 mm the accuracy is better for manually measured distances, since a distance less than 5 mm is more difficult to measure using the 3D automated software than for the 2D manual measurement. Indeed, using the automated software, only 3 patients had a distance lower than 5 mm against 12 manually measured. Therefore, the sensitivity in this case strongly suffered from the low number of patients in this group. These findings are plotted in the comparison charts between the ROC curves (Fig. 5).

**Table 2:** Diagnostic performance of the two different methods for computing tumour-to-nipple-areola complex distance at different cut-off values

Parameters	Automatic				Manual				*p* value
	Best (≤ 30 mm)	≤ 5 mm	≤ 10 mm	≤ 20 mm	Best (≤ 21 mm)	≤ 5 mm	≤ 10 mm	≤ 20 mm	
Sensitivity (%)	78.3	8.7	26.1	34.8	73.9	43.5	43.5	73.9	0.537
Specificity (%)	71.4	100.0	100.0	85.7	61.2	95.9	85.7	61.2	0.197
PPV (%)	56.3	100.	100.0	53.3	47.2	83.3	58.8	47.2	0.276
NPV (%)	87.5	70.0	74.3	73.7	83.3	78.3	76.4	83.3	0.477
Accuracy (%)	73.6	70.8	76.4	69.5	65.3	79.2	72.2	65.3	0.281

*PPV* positive predictive value, *NPV* negative predictive value

Considering the 20 mm cut-off, which is currently the most widely used in clinical practice, the sensitivity of the automated method was significantly higher than that of the manual method (*p* < 0.001). However, the manual method showed a lower specificity than the automated method (*p* < 0.001). At the bottom line, the difference between the two methods as regards PPV (*p* = 0.471) and PNV (*p* = 0.164) was not significant.

The results obtained with our dataset have been compared with those we have previously obtained with the same 3D method on images obtained on a different setting, as shown in Table 3.

**Table 3:** Comparison between the diagnostic performance obtained in the current study and those obtained in the previous study by Giannini et al. [16]

Index	Current study (best cut-off ≤ 30 mm)	Giannini et al. [16] (best cut-off ≤ 21 mm)
Sensitivity (%)	78	72
Specificity (%)	72	80
Positive predictive value (%)	56	56
Negative predictive value (%)	88	89
Accuracy	74	78

## Discussion

Preoperative MRI has been shown to have a crucial role in the assessment of breast cancer patients potentially eligible for NSM. Compared to mammography, which is a 2D imaging modality, breast MRI provides a 3D evaluation of the whole breast, thus reducing the loss of spatial information about tumour extent and location. For that reason, MRI has been considered as the method of choice to preoperatively predict occult nipple involvement [20, 21].

To facilitate surgical planning and to standardise the use of tumour-to-NAC distance as the main predictor of nipple infiltration by tumour, Giannini et al. [16] recently developed an automated method to compute the 3D tumour-to-NAC distance, which overcomes the performance of manual 2D methods in predicting NAC involvement. However, this method was developed and validated using images acquired with the same MRI scanner and having the same acquisition protocol. When developing automated methods, this could represent a strong bias, since images strongly differ between scanners and imaging protocols.

In the current study, we validated this algorithm using an external dataset of images acquired in a different centre, using a different MRI scanner and acquisition protocol from that used in the development phase. The performance reached by this method with this external dataset (sensitivity 78%, specificity 72%) demonstrated that this 3D automated method could represent a reliable method to preoperatively compute tumour-to-NAC distance, improving the management of patients candidate for NSM. The algorithm had a failure rate of only 5% because of the failure of the nipple (two cases) or tumour (two cases) segmentation.

The automated system presented in this study may show many advantages. First, the 3D tumour-to-NAC measurements were more reliable than the 2D measurements calculated using MIP images. In fact, when a measurement is carried out on the axial/sagittal projection, the information along the *z*-/*y*-axes is lost, with the consequent chance that the lesion and the nipple appear closer, as lying on the same *x*-/*y*-axis. Actually, the lesion and nipple are often more distant, as they are seated in different slices of MRI volume. In fact, the distance calculated by the automated algorithm was greater than that manually calculated in 55/72 cases (76%). In a recent study [21], the issue of three-dimensional “real” distance has been discussed. However, in that case, the measurements were done in a completely manual way, by computing four distinct distances in each case, using digital images on flat-screen liquid-crystal display monitors. This is a time-consuming task, which is difficult to apply in clinical practice. In addition, this study [21] did not make a comparison with the standard methods.

Interestingly, no fully automated methods for the nipple segmentation on MRI are available yet. The nipples often differ in form and intensity of the signal in different patients; in addition, the nipple is not always perfectly located at the centre of the T2-weighted image and, when inverted, cannot always be distinguished from glandular tissue. In our experience, 92% of the nipples were properly segmented by the algorithm.

Taken for granted that the tumour-to-NAC distance is up to date the most useful parameter for the preoperative assessment when NSM is under consideration, the main issue is to define the best cut-off value capable of predicting NAC involvement. In this regard, the literature is inhomogeneous. Some authors propose 10 mm as the ideal cut-off [13], while others recommend 20 mm [22, 23]. In a recent study, a distance of 5 mm was suggested [14].

In the present series, the 3D automated method improved the diagnostic performance when compared to 2D manual measurement, even though not significantly. In particular, the best compromise between sensitivity and specificity for each method was reached using the cut-off of 30 mm for the automated method and of 21 mm for the manual method. This difference is consistent with the increase by 11.5 mm in the average tumour-to-NAC distance when processed by the automated method versus the manual one and with the previously mentioned greater distance in 76% of cases as compared to the manual measurement.

As shown in Table 2, specificity and PPV of the automated method overcome those of the manual method at all the cut-off values. Sensitivity and NPV are instead higher only for the best cut-off (30 mm) since the automated method is not able to clearly identify NAC-negative patients at smaller distances. All the patients with tumour-to-NAC automated distance ≤ 5 mm and ≤ 10 mm showed tumour involvement of the nipple at the final pathology, confirming the high specificity of the automated method. This performance is higher compared to the manual measurement, which showed 96% and 86% specificity at these thresholds. However, the sensitivity at ≤ 5 and ≤ 10 mm was very low: only 9% and 26% of the patients with tumour involvement of the nipple were positive when tested with the automated method at these cut-off values, respectively.

Since the aim of the assessment before surgery is to propose NSM to all patients who may potentially preserve the nipple (i.e. patients without NAC tumour involvement at pathology), specificity and PPV are the most useful preliminary parameters to know. As a high specificity is related to a low sensitivity, many patients with NAC involvement (i.e. patients with NAC tumour involvement at pathology) will still be candidates for NSM.

By choosing the cut-off at ≤ 10 mm, the high specificity (100%) allows to exclude all the patients who certainly will not be able to keep the nipple, while the low sensitivity (26%) causes the inclusion in the selection for NSM of most patients (74%) with NAC involvement. However, the current protocol for the NSM mandates the intraoperative histological examination of the retroareolar tissue, which shows a good negative predictive ability. In such cases, the surgery may be converted to SSM at the same surgical time.

The data obtained from our study are similar to those we have previously obtained with the same 3D methods on images obtained on a different equipment, as shown in Table 3, as expected from an automated not operator-sensitive and therefore more reliable algorithm. The main difference is between the value of the 3D best cut-off considered in the previous and in the present study, 21 mm and 30 mm, respectively. This is an important issue that deserves further investigation. A prospective series taking into account the breast volume as well as the use of a standardised MRI protocol for the acquisition of T2-weighted and DCE images may help overcoming the gap.

**Fig. 6:**
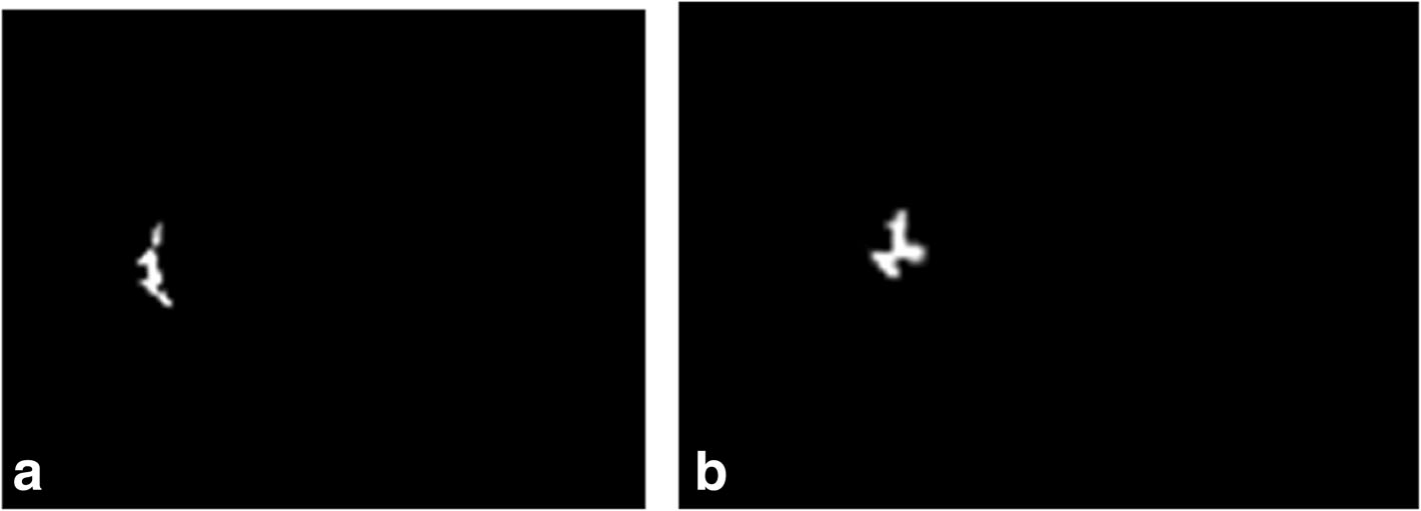
Example of nipple masks not perfectly segmented by the algorithm. **a** Example 1. **b** Example 2

Limitations of our study are mainly related to its retrospective design. Our series covers a period of almost 6 years, during which there have been technological advances that, although modest, could have influenced the signal to noise ratio and the image quality, changing the segmentation capabilities (especially for the nipples) of the automated algorithm, as shown in Fig. 6. The incomplete segmentation of the NAC causes the automated distance to be computed between the edge of the lesion and the front part of the nipple, rather than between the edge of the lesion and the base of NAC. Moreover, the correct localisation of the nipple could be difficult in some cases, such as malposition or introflexion, and need the supervision of a technician with specialised experience in breast MRI. The implementation of this step is definitely needed to maximise the accuracy of the algorithm. Secondly, choosing the cut-off at ≤ 10 mm, the sensitivity of the automated method remains low and many positive cases will be overlooked. It is, therefore, necessary to perform the frozen examination of subareolar tissue during surgery, which is currently considered the safest method to predict nipple involvement.

In conclusion, our study suggests that breast MRI is a promising method for the preoperative assessment of patients candidate for NSM by predicting the occult involvement of the NAC. Our novel 3D automated method seems to improve the results obtained with the 2D manual distance measurement also being validated on an independent external dataset. A cut-off value of ≤ 10 mm provided great accuracy as all the patients with a tumour-to-NAC distance ≤ 10 mm require the removal of the nipple. If integrated into clinical practice, this method could be useful to reduce the variability in selecting patients who may have the nipple preserved.

## Data Availability

The datasets used and/or analysed during the current study are available from the corresponding author on reasonable request.

## Abbreviations

AUC: Area under the curve
DCE: Dynamic contrast-enhanced
MIP: Maximum intensity projection
MRI: Magnetic resonance imaging
NAC: Nipple-areola complex
NSM: Nipple-sparing mastectomy
ROC: Receiver operating characteristic
SSM: Skin-sparing mastectomy

## References

[1] Toth BA, Lappert P (1991). Modified skin incisions for mastectomy: the need for plastic surgical input in preoperative planning. Plast Reconstr Surg.

[2] Didier F, Radice D, Gandini S (2009). Does nipple preservation in mastectomy improve satisfaction with cosmetic results, psychological adjustment, body image and sexuality?. Breast Cancer Res Treat.

[3] Laronga C, Kemp B, Johnston D, Robb GL, Singletary SE (1999). The incidence of occult nipple-areola complex involvement in breast cancer patients receiving a skin-sparing mastectomy. Ann Surg Oncol.

[4] Stanec Z, Žic R, Budi S (2014). Skin and nipple-areola complex sparing mastectomy in breast cancer patients: 15-year experience. Ann Plast Surg.

[5] National Comprehensive Cancer Network (2018) NCCN clinical practice guidelines in oncology. 2016 Breast Cancer. Version:1 https://www.nccn.org/professionals/physician_gls/default.aspx 10.6004/jnccn.2018.0006 29439178

[6] Metcalfe KA, Cil TD, Semple JL (2015). Long-term psychosocial functioning in women with bilateral prophylactic mastectomy: does preservation of the nipple-areolar complex make a difference?. Ann Surg Oncol.

[7] Petit JY, Veronesi U, Orecchia R (2012). Risk factors associated with recurrence after nipple-sparing mastectomy for invasive and intraepithelial neoplasia. Ann Oncol.

[8] De La Cruz L, Moody AM, Tappy EE (2015). Survival, disease-free survival, local recurrence, and nipple-areolar recurrence in the setting of nipple-sparing mastectomy: a meta-analysis and systematic review. Ann Surg Oncol.

[9] Peled AW, Wang F, Foster RD (2016). Expanding the indications for total skin-sparing mastectomy: is it safe for patients with locally advanced disease?. Ann Surg Oncol.

[10] Schecter AK, Freeman MB, Giri D (2006). Applicability of the nipple-areola complex-sparing mastectomy: a prediction model using mammography to estimate risk of nipple-areola complex involvement in breast cancer patients. Ann Plast Surg.

[11] Loewen MJ, Jennings JA, Sherman SR (2008). Mammographic distance as a predictor of nipple-areola complex involvement in breast cancer. Am J Surg.

[12] Zhang H, Li Y, Moran MS (2015). Predictive factors of nipple involvement in breast cancer: a systematic review and meta-analysis. Breast Cancer Res Treat.

[13] D’Alonzo M, Martincich L, Biglia N (2012). Clinical and radiological predictors of nipple-areola complex involvement in breast cancer patients. Eur J Cancer.

[14] Ponzone R, Maggiorotto F, Carabalona S (2015). MRI and intraoperative pathology to predict nipple-areola complex (NAC) involvement in patients undergoing NAC-sparing mastectomy. Eur J Cancer.

[15] Giannini V, Vignati A, Morra L (2010). A fully automatic algorithm for segmentation of the breasts in DCE-MR images. Conf Proc IEEE Eng Med Biol Soc.

[16] Giannini V, Bianchi V, Carabalona S (2017). MRI to predict nipple-areola complex (NAC) involvement: an automatic method to compute the 3D distance between the NAC and tumor. Journal of Surgical Oncology.

[17] Sardanelli F, Boetes C, Borisch B (2010). Magnetic resonance imaging of the breast: recommendations from the EUSOMA working group. Eur J Cancer.

[18] Tustison NJ, Avants BB (2013) Explicit B-spline regularization in diffeomorphic image registration. Front Neuroinform 7. 10.3389/fninf.2013.00039 10.3389/fninf.2013.00039 PMC3870320 24409140

[19] Vignati A, Giannini V, De Luca M (2011). Performance of a fully automatic lesion detection system for breast DCE-MRI. J Magn Reson Imaging.

[20] Piato JR, de Andrade RD, Chala LF (2016). MRI to predict nipple involvement in breast cancer patients. AJR Am J Roentgenol.

[21] Byon W, Kim E, Kwon J (2014). Magnetic resonance imaging and clinicopathological factors for the detection of occult nipple involvement in breast cancer patients. J Breast Cancer.

[22] Moon JY, Chang YW, Lee EH (2013). Malignant invasion of the nipple-areolar complex of the breast: usefulness of breast MRI. AJR Am J Roentgenol.

[23] Garcia-Etienne CA, Cody Iii HS, Disa JJ (2009). Nipple-sparing mastectomy: initial experience at the Memorial Sloan-Kettering Cancer Center and a comprehensive review of literature. Breast J.

